# Antifungal Potential of Synthetic Peptides against *Cryptococcus neoformans*: Mechanism of Action Studies Reveal Synthetic Peptides Induce Membrane–Pore Formation, DNA Degradation, and Apoptosis

**DOI:** 10.3390/pharmaceutics14081678

**Published:** 2022-08-12

**Authors:** Tawanny K. B. Aguiar, Nilton A. S. Neto, Cleverson D. T. Freitas, Ayrles F. B. Silva, Leandro P. Bezerra, Ellen A. Malveira, Levi A. C. Branco, Felipe P. Mesquita, Gustavo H. Goldman, Luciana M. R. Alencar, Jose T. A. Oliveira, Ralph Santos-Oliveira, Pedro F. N. Souza

**Affiliations:** 1 Department of Biochemistry and Molecular Biology, Federal University of Ceará, Fortaleza 60451-970, CE, Brazil; 2 Department of Fisheries Engineering, Federal University of Ceará, Fortaleza 60455-970, CE, Brazil; 3 Drug Research and Development Center, Department of Physiology and Pharmacology, Federal University of Ceará, Fortaleza 60430-275, CE, Brazil; 4 Faculty of Pharmaceutical Sciences of Ribeirão Preto, University of São Paulo, São Paulo 14040-903, SP, Brazil; 5 Department of Physics, Laboratory of Biophysics and Nanosystems, Federal University of Maranhão, São Luís 65080-805, MA, Brazil; 6 Laboratory of Nanoradiopharmaceuticals and Radiopharmacy, Zona Oeste State University, Rio de Janeiro 23070-200, RJ, Brazil; 7 Brazilian Nuclear Energy Commission, Nuclear Engineering Institute, Rio de Janeiro 21941-906, RJ, Brazil

**Keywords:** synthetic antifungal peptides, *Cryptococcus neoformans*, cryptococcal meningitis, inhibition, apoptosis induction

## Abstract

*Cryptococcus neoformans* is a human-pathogenic yeast responsible for pneumonia and meningitis, mainly in patients immunocompromised. Infections caused by *C. neoformans* are a global health concern. Synthetic antimicrobial peptides (SAMPs) have emerged as alternative molecules to cope with fungal infections, including *C. neoformans*. Here, eight SAMPs were tested regarding their antifungal potential against *C. neoformans* and had their mechanisms of action elucidated by fluorescence and scanning electron microscopies. Five SAMPs showed an inhibitory effect (MIC_50_) on *C. neoformans* growth at low concentrations. Fluorescence microscope (FM) revealed that SAMPs induced 6-kDa pores in the *C. neoformans* membrane. Inhibitory assays in the presence of ergosterol revealed that some peptides lost their activity, suggesting interaction with it. Furthermore, FM analysis revealed that SAMPs induced caspase 3/7-mediated apoptosis and DNA degradation in *C. neoformans* cells. Scanning Electron Microscopy (SEM) analysis revealed that peptides induced many morphological alterations such as cell membrane, wall damage, and loss of internal content on *C. neoformans* cells. Our results strongly suggest synthetic peptides are potential alternative molecules to control *C. neoformans* growth and treat the cryptococcal infection.

## 1. Introduction

Fungi cause various diseases, from mild superficial mycoses on the skin to severe invasive lung infections. Fungal infections can cause more than 50% mortality, especially those caused by fungi from genus *Cryptococcus*, *Candida*, *Aspergillus*, and *Pneumocystis*, among the most lethal human pathogens [1,2,3]. The ability to treat those infections was revolutionary to medicine. However, in the past 80 years, fungi have become resistant to most or even all available antifungal agents. Thus, this creates an urgent need for new molecules to overcome the resistance and develop novel treatments for fungal infectious diseases [2,4].

Among those human-pathogenic fungi, *C. neoformans* is a yeast responsible for pneumonia and meningitis. *C. neoformans* is mostly common in immunocompromised patients, organ transplanted patients, those submitted to cancer chemotherapy, and HIV+ patients [5]. *Cryptococcus* strains have evolved virulence traits that make it a unique and highly resistant fungal pathogen, such as a capsule, which protects against phagocytosis [6]. In addition, the inappropriate use of antifungal agents accelerated the development of antifungal resistance. The resistance of *C. neoformans* to echinocandins, the newest fungicidal drug class, is a huge problem, leading public health systems worldwide without an option to treat *C. neoformans* infections [7]. This has driven the need for new molecules to cope with *C. neoformans* infection and develop new therapies [8,9,10]. Therefore, seeking and developing new molecules effective against *C. neoformans* is imperative to produce a new drug to treat cryptococcal infections. Several research groups worldwide have been seeking new alternatives to overcome the threat imposed by *C. neoformans* [9,10].

Thus, searching for new compounds with different mechanisms than conventional drugs to inhibit fungal growth is urgently required. Recently, two sequential studies showed the potential of Ellagic acid (EA), a polyphenolic compound present in plants, which has presented high activity against *C. neoformans* [9,10]. In the first study, the authors showed that mice treated with EA presented a survival rate of 70% toward *C. neoformans* infection. In contrast, mice treated with fluconazole had only 20% survival [9]. In the second study [10], it was shown in silico and in vitro that EA interacts and inhibits the laccase from *C. neoformans*, thus indicating that laccase is an excellent target to focus on the treatment of *C. neoformans* infection. Antimicrobial peptides are also potential alternative molecules. For instance, Mahindra et al. [11] reported many natural peptides that present activity against *C. neoformans*. However, some natural peptides presented in the study were toxic to kidney cells, making them inappropriate for further studies. The natural antimicrobial peptides presented several problems to clinical application. Usually, the natural peptides are toxic to human cells, are fairly susceptible to proteolysis, and have a high production cost [12].

In this scenario, synthetic antimicrobial peptides (SAMPs) are a potential alternative to overcoming infections caused by *C. neoformans*. Compared to natural peptides, SAMPs are rationally designed to enhance their antimicrobial activity and remove limitations of natural peptides (e.g., toxicity) [12]. This makes SAMPs good candidates for new antifungal drugs. For example, our research group has designed eight synthetic peptides that present no toxicity to human erythrocytes, fibroblast, keratinocytes, and even zebrafish embryos, in addition, to being resistant to proteolysis [12,13].

Therefore, it was hypothesized that synthetic antimicrobial peptides, which had no toxicity as discussed before, have antifungal activity against *C. neoformans* by damaging cell membranes, making the development of resistance hard. Based on that, here, these eight are bioinspired from three antimicrobial plant proteins, *Mo*-CBP3 (SAMPs named *Mo*-CBP_3_-PepI, *Mo*-CBP_3_-PepII, and *Mo*-CBP_3_-PepIII) from *Moringa oleifera* seeds [14]. *Rc*-2S-Alb (SAMPs named *Rc*Alb-PepI, *Rc*Alb-PepII, and *Rc*Alb-PepIII) [12] from *Ricinus communis*. Chitinase (SAMPs named PepGAT and PepKAA) [15] from *Arabidopsis thaliana* with proven antifungal, antibacterial, antibiofilm, and antiviral activity against different human pathogens [12,13] had their antifungal potential tested against *C. neoformans*. Of the eight, five SAMPs presented great inhibitory activity against *C. neoformans*, and investigation of the mechanism of action revealed those peptides targeted cell membrane and induced *C. neoformans* apoptosis. The great importance of this work is that synthetic peptides, which are not toxic, present activity against *C. neoformans* and can potentially be used in developing new drugs to overcome *C. neoformans* resistance to drugs.

## 2. Materials and Methods

### 2.1. Fungal Strains, Chemicals, and Synthetic Peptides

*Cryptococcus neoformans* (ATCC 32045) was gently provided by the Department of Pathology of the Federal University of Ceará (UFC), Fortaleza, Brazil. All the chemicals used in the experiments were obtained from Sigma Aldrich (São Paulo, SP, Brazil).

### 2.2. Peptide Synthesis

The synthetic peptides Mo-CBP3-PepI, Mo-CBP3-PepII, Mo-CBP3-PepIII, RcAlb-PepI, RcAlb-PepII, RcAlb-PepIII, PepGAT, and PepKAA were chemically synthesized by the company Chempeptide (Shanghai, China). The quality and purity (≥95%) were analyzed by reverse-phase high-performance liquid chromatography (RP-HPLC, Jasco, Easton, MD, USA) and mass spectrometry (Waltham, MA, USA).

### 2.3. The Minimum Inhibitory Concentration Assay

The minimum inhibitory concentration (MIC) assay was performed using a broth microdilution test using 96-well plates [12]. The cells were grown on YPD agar for approximately 15 days, and subsequently, cryptococcal cells were resuspended in YPD medium and standardized at 10^6^ cells mL^−1^. In 96-well plates, 25 µL of YPD with cryptococcal cells and 25 µL of each synthetic peptide at different concentrations (50 to 0.004 µg mL^−1^) were added. The microplates were incubated for 24 h. Then, the absorbance was measured at 600 nm using an automated microplate reader (Epoch, Biotek, Santa Clara, CA, USA). The negative control for inhibition of *C. neoformans* was 5% DMSO. The positive control for inhibition was made by two antifungal drugs, Nystatin (NYS, 1000 µg mL^−1^) and Itraconazole (ITR, 1000 µg mL^−1^).

### 2.4. Mechanisms of Action Employed by Peptides

#### 2.4.1. Ergosterol Interaction Assay

The interaction of peptides with ergosterol was evaluated following [16]. Cryptococcal cells (10^6^ cells mL^−1^) and ergosterol (0.02, 0.04, and 0.08 mg mL^−1^), *Mo*-CBP_3_-PepII, *Rc*Alb-PepII, *Rc*Alb-PepIII, PepGAT and PepKAA (MIC50) and YPD medium (1:1:1:1 *v*/*v*) were incubated in 96-well plates at 30 °C for 24 h. Next, fungal growth was measured at 620 nm using an automated microplate reader. DMSO-NaCl and Nystatin were used as controls.

#### 2.4.2. Cell Membrane Integrity Assay

To evaluate the pore formation induced by peptides on the *C. neoformans* membrane, the methodology described by Dias et al. [12] was used. After the antifungal assay (under the same conditions described above), the samples were washed with 0.15 M NaCl, centrifuged (5000× *g* 5 min at 4 °C), and incubated with PI at 1 μM for 30 min at room temperature in the dark. After that, the cells were analyzed under a fluorescence microscope (Olympus System BX60, Olympus, Tokyo, Japan) with an excitation wavelength of 488 nm and an emission wavelength of 525 nm. Fluorescent *C. neoformans* cells were counted using ImageJ software using the Cell Counter plugin [17]. The same picture has the cells counted in the bright and fluorescent field. The number of cells in the bright field was considered 100%. In the fluorescent field, cells that released fluorescent were called positive cells, and those without fluorescence were called negative cells. The following equation reached the % of positive cells: Number of positive cells × 100/number of cells in bright field. The number of negative cells was calculated following the equation: number of cells in the bright field—number of positive cells. Three different images were used to count cells.

Additionally, the methodology described by Dias et al. [12] was used to evaluate the size of pores formed. The cells of *C. neoformans* were treated as above and incubated with 10 μM of conjugated fluorescein isothiocyanate (FITC)-Dextran with 6 kDa (Sigma Aldrich, São Paulo, SP, Brazil). After incubation for 30 min at 25 °C in the dark, the cells were washed as above and observed under a fluorescence microscope (Olympus System BX60) with an excitation wavelength of 490 nm and emission wavelength of 520 nm. Fluorescent *C. neoformans* cells were counted using ImageJ software using the Cell Counter plugin [17]. The same picture has the cells counted in the bright and fluorescent fields, and the number of cells in the bright field was considered 100%. In the fluorescent field, cells that released fluorescent were called positive cells, and those without fluorescence were called negative cells. The following equation reached the % of positive cells: Number of positive cells × 100/number of cells in bright field. The number of negative cells was calculated following the equation: number of cells in the bright field—number of positive cells. Three different images were used to count cells.

#### 2.4.3. DNA Degradation on *C. neoformans* Induced by Peptides

The kit DeadEnd™ Fluorometric TUNEL System (Promega, São Paulo, SP, Brazil) followed the manufacturer’s instructions to evaluate the DNA degradation induced by peptides. Fluorescent *C. neoformans* cells were counted using ImageJ software using the Cell Counter plugin [17] described above in the cell membrane assay.

#### 2.4.4. Caspase 3/7 Assay

The caspase activity was measured after cell incubation for 24 h, in the presence and absence of synthetic peptides, according to the methodology described by Qorri and Harless [18], with some modifications. The cells were treated as above and then incubated using a 3 μL CellEvent^®^ reagent (ThermoFisher, São Paulo, SP, Brazil) for 30 min in the dark. Afterwards, cells were washed and centrifuged as mentioned above. Finally, the cells were observed under a fluorescence microscope (Olympus System BX60) with an excitation wavelength of 342 nm and an emission wavelength of 441 nm. Fluorescent C. neoformans cells were counted using ImageJ software using the Cell Counter plugin [17]. The same picture has the cells counted in the bright and fluorescent field. The number of cells in the bright field was considered 100%. In the fluorescent field, cells that released fluorescent were called positive cells, and those without fluorescence were called negative cells. The following equation reached the % of positive cells: Number of positive cells × 100/number of cells in bright field. The number of negative cells was calculated following the equation: number of cells in the bright field—number of positive cells. Three different images were used to count cells.

#### 2.4.5. Scanning Electron Microscopy (SEM)

The cells of *C. neoformans* untreated and treated with peptides were prepared and analyzed by SEM following Staniszewska et al. [19], with some adaptions. After the antifungal assay described previously in Section 2.3, cells were fixed with 1% (*v*/*v*) glutaraldehyde in 0.15 M sodium phosphate buffer at pH 7.2 for 16 h. Next, the cells were washed with sodium phosphate buffer at pH 7.2 and centrifuged (5000× *g* for 5 min at 4 °C) each time. Then, samples were dehydrated with increased ethanol concentrations (30%, 50%, 70%, 100%, and 100% [*v*/*v*]) for 10 min each at 25 °C and centrifuged as above each time. The final dehydration was performed with 50% (*v*/*v*) hexamethyldisilane (HDMS, Sigma, St. Louis, MI, USA) diluted in ethanol for 10 min, centrifuged as above, and then dehydrated with 100% HDMS. The dried cells were placed into a cover glass and covered with gold using a coating machine (Emitech-Q150TES, Quorum Technologies, Lewes, England) coupled with positron-emission tomography (PET). SEM analysis ran in a scanning electron microscope (Quanta 450 FEG, FEI, Waltham, MA, USA) with a magnification of 20,000×.

### 2.5. Statistical Analysis

All experiments were performed three times independently, and the values are expressed as the mean ± standard error. GraphPad Prism 5.01 (GraphPad Software company, Santa Clara, CA, USA) for Microsoft Windows was used to run the statistical analyses. All data obtained in the assays were submitted to ANOVA, followed by the Tukey test (*p* < 0.05).

## 3. Results

### 3.1. Antifungal Activity

Five of eight peptides were tested in 12 serial dilutions to reach the Minimum Inhibitory Concentration of peptides required to inhibit 50% of yeast growth (MIC_50_) (Table 1), *Mo*-CBP_3_-PepII, *Rc*Alb-PepII, *Rc*Alb-PepIII, PepGAT, and PepKAA presented an MIC_50_, respectively, of 25, 0.04, 0.04, 0.04, and 0.04 μg mL^−1^ (Table 1). Although Mo-CBP3-PepI, Mo-CBP3-PepIII, and RcAlb-PepI inhibited *C. neoformans* growth, none of them reached 50% of inhibition in all concentrations tested (Table 1). The peptides that reached the MIC_50_ were chosen to investigate the mechanism of action.

### 3.2. Ergosterol Interactions

**Figure 1:**
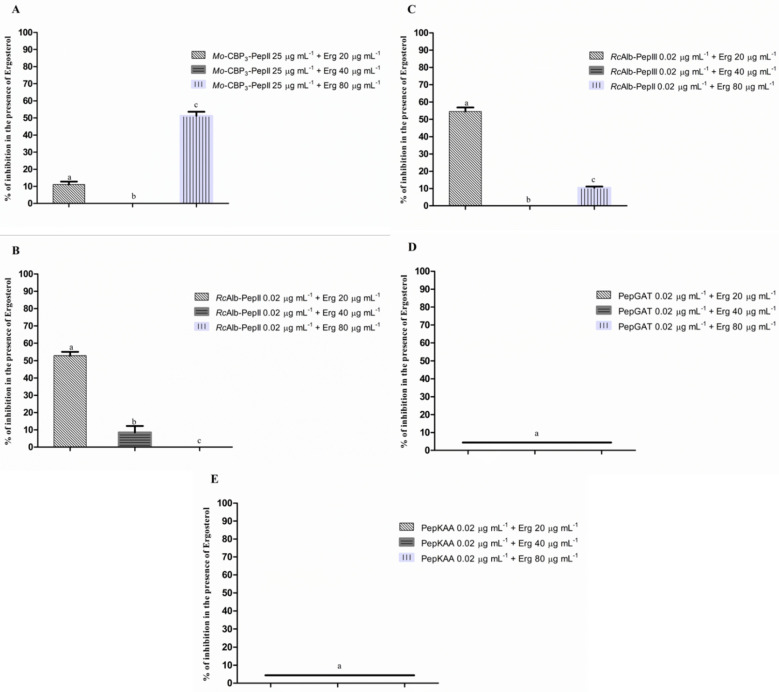
The interaction of ergosterol and its role in the antifungal activity of synthetic peptides. (**A**) *Mo*-CBP_3_-PepII, (**B**) *Rc*Alb-PepII, (**C**) *Rc*Alb-PepIII, (**D**) PepGAT, and (**E**) PepKAA DMSO-NaCl was used as a negative control. Data are presented as mean ± standard deviation (SD). Using the Tukey test, different letters represent the statistical difference (*p* < 0.05). The experiment was repeated three times.

We further investigated if peptides could interact with ergosterol in the membrane of C. neoformans. To evaluate that, peptides at MIC_50_ concentration were assayed against *C. neoformans* in the presence of ergosterol at concentrations of 20, 40, and 80 µg mL^−1^. To some extent, all peptides had antifungal activity against *C. neoformans* affected by the presence of ergosterol (Figure 1). For instance, *Mo*-CBP_3_-PepII and *Rc*Alb-PepIII completely lost their activity against *C. neoformans* in the presence of ergosterol at 40 µg mL^−1^ (Figure 1A,C). In contrast, *Rc*Alb-PepII lost activity in the presence of 80 µg mL^−1^ of ergosterol (Figure 1B). PepGAT and PepKAA were the most affected by the presence of ergosterol, losing the inhibitory activity in all tested concentrations (Figure 1D,E).

### 3.3. Membrane Pore Formation

**Figure 2:**
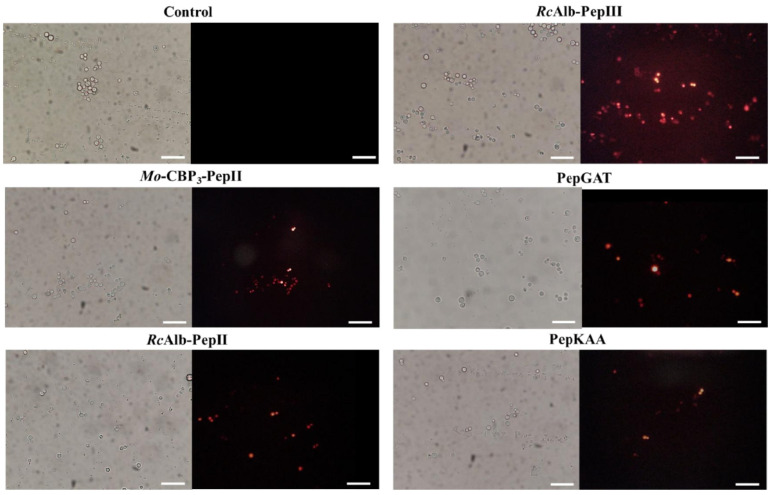
Fluorescence images showing membrane pore formation induced by *Mo*-CBP_3_-PepII, *Rc*Alb-PepII, *Rc*Alb-PepIII, PepGAT, and PepKAA, respectively, at 25, 0.04, 0.04, 0.04, and 0.04 μg mL^−1^. Detection of red fluorescence in the peptide-treated cells indicates that PI was internalized. In control (DMSO-NaCl), the absence of PI fluorescence indicates the integrity of the cell membrane. Bars: 100 µm.

The presence of pores on *C. neoformans* membranes was confirmed by propidium iodide (PI) uptake assay. PI interacts with DNA releasing red fluorescence but cannot pass through a healthy membrane. As expected, the healthy membrane of control *C. neoformans* did not allow the passage of PI and had no red fluorescence (Figure 2). In contrast, the PI uptake and red fluorescence showed that all peptides could induce pore formation on the *C. neoformans* membrane (Figure 2). 

**Figure 3:**
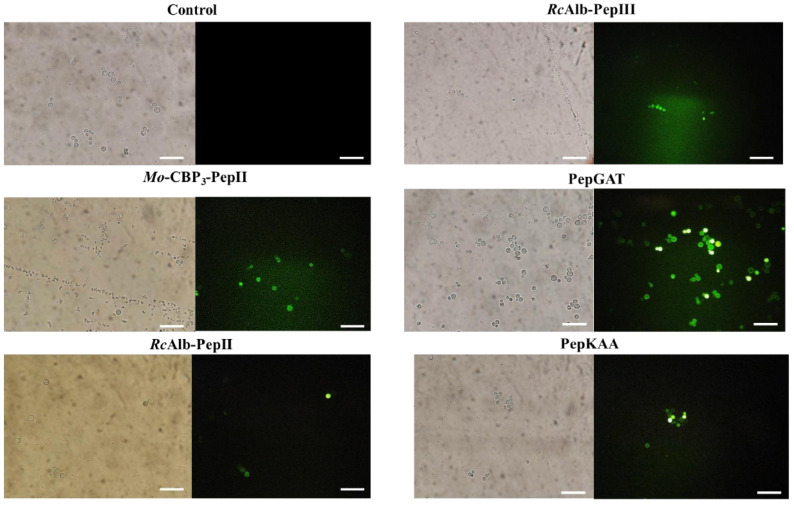
Fluorescence images showing membrane pore formation using fluorescein isothiocyanate (FITC)-dextran (6 kDa) induced by *Mo*-CBP_3_-PepII, *Rc*Alb-PepII, *Rc*Alb-PepIII, PepGAT, and PepKAA, respectively, at 25, 0.04, 0.04, 0.04, and 0.04 μg mL^−1^. *C. neoformans* cells were incubated with peptides and DMSO-NaCl. Detection of green fluorescence indicates that cells internalized FITC-dextran. Bars: 100 µm.

PI assay only provides information about the pores on the membrane without any clue about their size. An additional experiment using dextran with a size of 6 kDa conjugated with FITC (Fluorescein isothiocyanate—green fluorescence, Figure 3) revealed all peptides induced pore with a size of at least 6 kDa, which allows the movement of dextran by the membrane of *C. neoformans*.

### 3.4. DNA Degradation and Apoptosis in C. neoformans Cells Induced by Peptides

**Figure 4:**
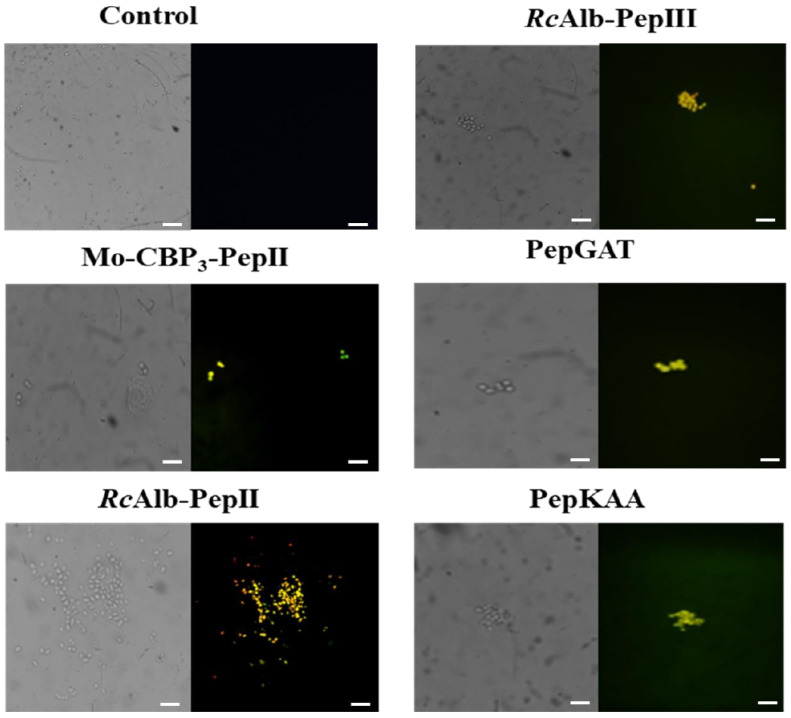
DNA fragmentation in *C. neoformans* cells induced by *Mo*-CBP_3_-PepII, *Rc*AlbPepII, *Rc*Alb-PepIII, PepGAT, and PepKAA, respectively, at 25, 0.04, 0.04, 0.04, and 0.04 μg mL^−1^. The cells were treated with peptides, and the control negative was DMSONaCl. Bars: 100 µm.

The *C. neoformans* cells treated with synthetics peptides at MIC_50_ concentration showed yellow fluorescence, indicating DNA degradation and fragmentation induced by peptides. The same was not observed in the control (Figure 4). 

**Figure 5:**
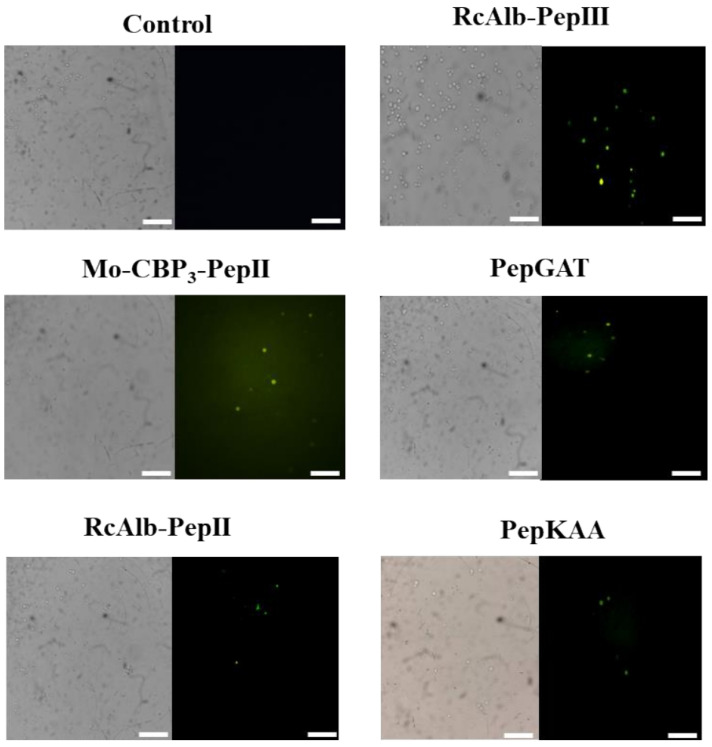
Apoptosis induction in *C. neoformans* cells by *Mo*-CBP_3_-PepII, *Rc*Alb-PepII, *Rc*Alb-PepIII, PepGAT, and PepKAA, respectively, at 25, 0.04, 0.04, 0.04, and 0.04 μg mL^−1^. Green fluorescence images show that treatment with the peptides (MIC_50_) activates caspases involved in programmed cell death. Control: DMSO-NaCl. Bars: 100 µm.

As peptides induced DNA degradation in *C. neoformans*, we reasoned that the peptides could also induce apoptosis in *C. neoformans* cells. A Caspase-3/7 Green Detection kit evaluated the activity of caspases. The activity of caspase-3/7, which suggests that the cell is in apoptosis, was observed in all the cells treated with synthetics peptides (MIC_50_) (Figure 5). The control, as expected, was not observed in fluorescence. 

### 3.5. Counting Cells

**Figure 6:**
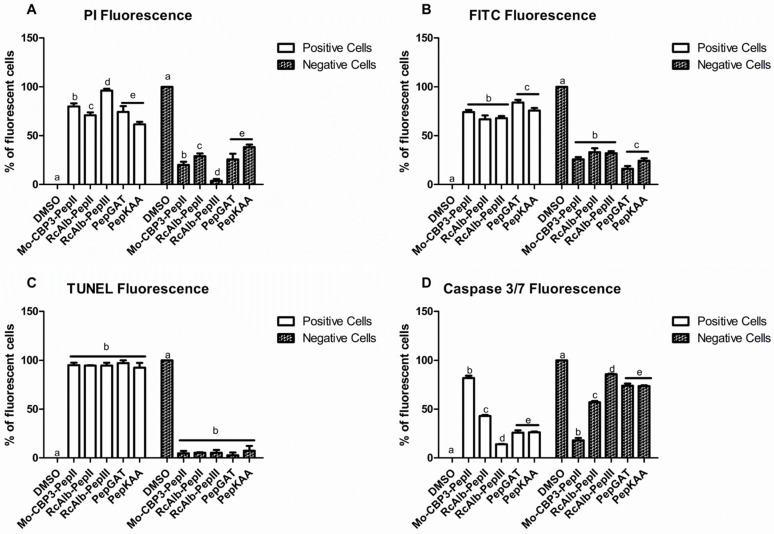
The number of fluorescent *C. neoformans* cells PI (**A**), Dextran-FITC (**B**), DNA fragmentation (**C**), and apoptosis (**D**). The letters represent the mean ± standard deviation of three replicates. Different lowercase letters indicate statically significant difference compared to DMSO-NaCl by analysis of variance (*p* < 0.05). The concentration of peptides was 25, 0.04, 0.04, 0.04, and 0.04 μg mL^−1^, respectively, for *Mo*-CBP_3_-PepII, *Rc*Alb-PepII, *Rc*Alb-PepIII, PepGAT, and PepKAA.

The counting of PI-fluorescent cells (positive cells) using the ImageJ program [17] revealed that 98% of cells treated with *Rc*Alb-PepIII presented a PI-fluorescence (Figure 6A). The evaluation of dextran-FITC-fluorescent cells using the ImageJ program revealed that after treatment with PepGAT and PepKAA, 86 and 85% of cells presented dextran-FITC-fluorescent (Figure 6B). The quantitative evaluation of TUNNEL-fluorescent cells revealed no difference in TUNNEL-fluorescent cells. All peptides induced fluorescence in around 97% of cells presented TUNNEL-fluorescence, indicating DNA damage after treatment (Figure 6C). The analysis on ImageJ program revealed *Mo*-CBP_3_-PepII was the most potent, with 90% of treated cells releasing fluorescence, indicating apoptosis establishment. All other peptides induced apoptosis to a different extent (Figure 6D).

### 3.6. SEM Analysis of C. neoformans Cells Morphology

**Figure 7:**
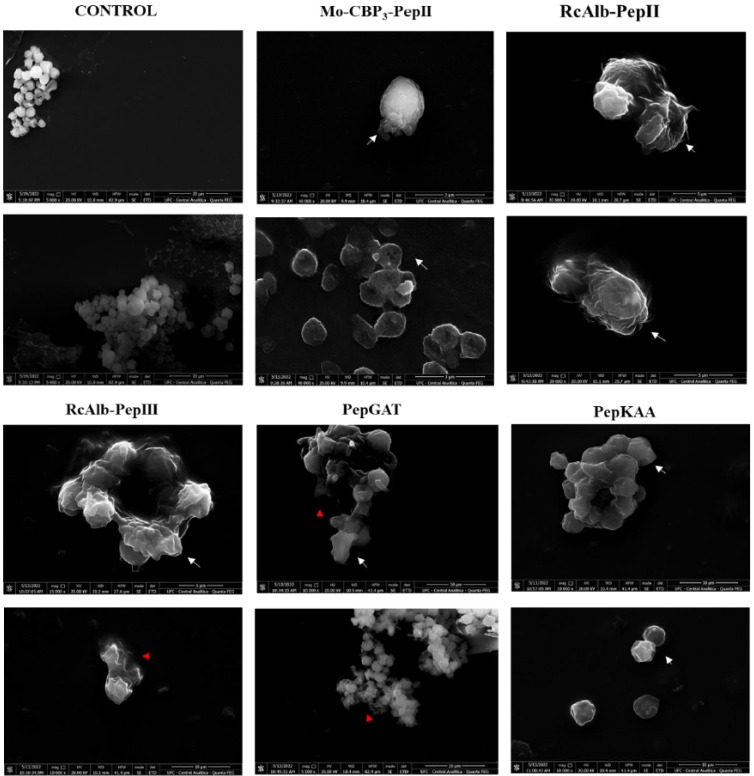
SEM images showing *C. neoformans* cells after treatment with *Mo*-CBP_3_-PepII, *Rc*Alb-PepII, *Rc*Alb-PepIII, PepGAT, and PepKAA, respectively, at 25, 0.04, 0.04, 0.04, and 0.04 μg mL^−1^ Control: DMSO-NaCl solution. White arrows show damage to cellular structure, and red arrows indicate cytoplasmic leakage.

SEM analysis was performed to evaluate the possible damage to *C. neoformans* morphology caused by synthetic peptides. SEM images revealed that control cells of *C. neoformans* are healthy with a standard spherical shape with the absence of any damage on a surface such as cracks and scars (Figure 7). In contrast, all peptides damaged *C. neoformans* cells (Figure 7). The peptides-treated cells presented deformation in cell morphology (Figure 7—white arrows), scars, cracks, broken cell wall, depression, distortion (Figure 7—white arrowhead), and loss of internal content (Figure 7 red arrowhead). The loss of internal content presented in SEM images confirmed the fluorescence assays (Figure 2 and Figure 3), showing damage to the membrane of *C. neoformans*.

## 4. Discussion

*C. neoformans* is a highly problematic echinocandin-resistant yeast, affecting immunocompromised patients such as transplanted patients, those under cancer chemotherapy, and HIV+. *C. neoformans* is responsible for a high number of fungal meningitis in immunocompromised patients and ∼15% of deaths in HIV+ patients each year [20,21]. In this sense, rationally designed synthetic peptides present high activity without the associated toxicity or susceptibility to proteolysis. In addition, the advances in chemical synthesis have made synthetic peptides application affordable [22]. Recently, a synthetic peptide AW9-Ma showed an MIC_50_ against *C. neoformans* at a concentration of 64 µg mL^−1^ [23], which is higher than all concentrations presented by the synthetic peptides (Table 1). However, the same peptide presented toxicity to human cells. Our previous works showed the peptides used here were not toxic to human erythrocytes (types A, B, and O), fibroblasts, and keratinocytes. Experiments also showed that peptides were not toxic to embryos of zebrafish. Additionally, our peptides prove to be resistant to proteolysis. Besides activity against *C. neoformans*, peptides must be considered as potential molecules against *C. neoformans* [12,13,14].

The peptides *Mo*-CBP_3_-PepII, *Rc*Alb-PepII, *Rc*Alb-PepIII, PepGAT, and PepKAA, presented here, reached an MIC_50_ against *C. neoformans* at low concentrations in addition to acting by multiple mechanisms of actions to damage *C. neoformans* cells. The minimum inhibitory concentration of peptides that inhibited 50% of *C. neoformans* growth was determined (Table 1). These concentrations defined for five peptides were used to study the mechanism of action employed by peptides against *C. neoformans*. For example, we provide an experiment showing that our peptides have their anti-cryptococcal activity affected when the cultures were supplied with different concentrations of exogenous ergosterol (Figure 1). The activity reduction strongly indicates that these peptides have a higher affinity for free-ergosterol than the ergosterol in the membrane of fungi. This result indicates that in normal conditions, in the absence of free ergosterol, peptides can target the ergosterol in the membrane of fungal cells.

The cell membrane is a complex system composed of many lipids, complete embedded or anchored proteins, and sterol types that act as stabilizers. In fungal membranes, ergosterol is the primary compound of sterols, making it the target of many antifungal drugs. For example, itraconazole targets the enzyme lanosterol 14-α-demethylase involved in ergosterol biosynthesis, blocking its production and leading to cell death [24].

We have shown that some of our peptides have an affinity for ergosterol. One of the consequences of the interaction of the peptides with ergosterol is the destabilization of the plasma membrane, often leading to pore formation. In literature, a work shows that an anticandida protein, *Mo*-CBP_2_, exerts its activity by interacting with ergosterol [16]. Nevertheless, to the best of our knowledge, no study discussed the anti-cryptococcal activity of synthetic peptides interacting with ergosterol. *Mo*-CBP_3_-PepII [14], *Rc*Alb-PepII and *Rc*Alb-PepIII [12], PepGAT, and PepKAA [15] proved experimentally that they can induce pore formation in other human pathogenic yeasts. This study was not different, as revealed by the PI influx assay (Figure 2).

Plasma membrane remodeling upon external insults is metabolically expensive [15]. To target and induce pore formation in membranes is not new for synthetic peptides. For instance, MSI-1, a synthetic cationic peptide, demonstrated membrane disruption by pore formation, resulting from its interaction with the membrane [25]. The ability to form pores in the membrane is not prevalent in all synthetic peptides. Still, it is an important mechanism, as they compromise the development of fungal resistance since changes in membrane composition can be dangerous for cellular life [15].

The PI uptake assay is a standard assay to show the membrane pore formation. However, it only indicates the presence of a tiny pore on the membrane, which sometimes does not necessarily result in cell death. To move forward on the damage to the membrane, we provide an experiment that revealed all peptides induced pore formation of size, at least 6 kDa (Figure 3). Different from the pore size that allows the movement of PI, a pore 6 kDa-sized on the membrane is a huge problem that cells have to deal with. A big pore allows the movement of small molecules and other peptides and proteins. The question arises: how do small synthetic peptides induce a pore of at least 6 kDa on *C. neoformans* membranes? We have a hypothesis that could explain that.

We suggest the big pore formed by peptides on the membrane is due to self-association ability once inserted into the membrane. Self-association is a vital characteristic of antimicrobial peptides and is responsible for the ability to induce pore formation [26]. With a big pore, we reasoned that those peptides could be internalized by an energy-independent mechanism and find a target in the cytoplasm. After reaching the cytoplasm, peptides could interfere in dozens of cellular pathways that drive cell death. For instance, Maurya et al. [27] described two synthetic peptides, VS2 and VS3, which can induce pore formation on *C. albicans* membranes and move through them, reaching the target on the cytoplasm.

In addition to membrane destabilization, the peptides were able to induce DNA fragmentation (Figure 4), followed by caspase-mediated programmed cell death (cmPCD) (Figure 5). These results are consonant because caspase-3 is responsible for initiating apoptosis by inducing DNA fragmentation [28]. Caspase-3 starts apoptotic DNA fragmentation by inactivating a protein called DNA fragmentation factor-45 (DFF45) and an inhibitor of caspase-activated DNase (ICAD). This releases a caspase-3-activated DNase, which starts DNA fragmentation.

We have four possible explanations for these sequential events involving DNA fragmentation and cmPCD. First, peptides interact with an unknown target outside the cell and trigger signaling inside the cell leading to DNA fragmentation and cmPCD. Second, these events were triggered by the damage caused by peptides on the cell membrane leading to cytoplasmic stress, and thus DNA fragmentation and cmPCD. Third, peptides move through the huge pore formed, and once inside the cell-induced, those events lead to cell death. Fourth, considering all this is a dynamic process, it is possible to suggest that all three explained above could happen simultaneously, giving no chance to *C. neoformans* cells to survive.

The SEM analysis corroborates all those damages caused by peptides to *C. neoformans* cells (Figure 6). Peptides-treated cells showed severe morphological alterations such as the loss of intracellular content, broken cell wall, and depression-like cavities in the cell, probably due to the pores caused by the interaction of the peptides in the membrane. Here, it was shown that synthetic peptides have different targets in potential as candidates in the treatment of fungal infections are fascinating, since the fungus would need to develop different strategies to resist.

## 5. Conclusions

Our results revealed that five synthetic antimicrobial peptides are active against *C. neoformans* at very low concentrations. Studies of mechanisms of action revealed those peptides damage the membrane and cell wall of *C. neoformans* cells and induce DNA fragmentation leading to apoptosis. These results revealed the peptides as alternative molecules to treat cryptococcal infection, with multiple mechanisms of action supporting anti-cryptococcal activity, making the development of resistance difficult. Based on the mechanisms employed by the peptides, it is feasible to suggest that peptides also have the potential to synergize drugs that are not effective anymore against *C. neoformans*.

## Figures and Tables

**Table 1:** Minimum inhibitory concentration (MIC) of synthetic against *C. neoformans*.

Peptides	MIC_50_ (µg mL^−1^) against *C. neoformans*
*Mo*-CBP_3_-PepI	^a^ ND
*Mo*-CBP_3_-PepII	25
*Mo*-CBP_3_-PepIII	ND
*Rc*Alb-PepI	ND
*Rc*Alb-PepII	0.04
*Rc*Alb-PepIII	0.04
PepGAT	0.04
PepKAA	0.04
Nystatin	250
Itraconazole	500

^a^ ND means that MIC_50_ was not reached.

## Data Availability

The data supporting this study’s findings are available on request from the corresponding author.
